# Reformulation of Consumer Health Queries with Professional Terminology: A Pilot Study

**DOI:** 10.2196/jmir.6.3.e27

**Published:** 2004-09-03

**Authors:** Robert M Plovnick, Qing T Zeng

**Affiliations:** ^1^Decision Systems GroupBrigham and Women's HospitalHarvard Medical SchoolBoston MAUSA

**Keywords:** Information retrieval, consumer informatics, Internet

## Abstract

**Background:**

The Internet is becoming an increasingly important resource for health-information seekers. However, consumers often do not use effective search strategies. Query reformulation is one potential intervention to improve the effectiveness of consumer searches.

**Objective:**

We endeavored to answer the research question: "Does reformulating original consumer queries with preferred terminology from the Unified Medical Language System (UMLS) Metathesaurus lead to better search returns?"

**Methods:**

Consumer-generated queries with known goals (n=16) that could be mapped to UMLS Metathesaurus terminology were used as test samples. Reformulated queries were generated by replacing user terms with Metathesaurus-preferred synonyms (n=18). Searches (n=36) were performed using both a consumer information site and a general search engine. Top 30 precision was used as a performance indicator to compare the performance of the original and reformulated queries.

**Results:**

Forty-two percent of the searches utilizing reformulated queries yielded better search returns than their associated original queries, 19% yielded worse results, and the results for the remaining 39% did not change. We identified ambiguous lay terms, expansion of acronyms, and arcane professional terms as causes for changes in performance.

**Conclusions:**

We noted a trend towards increased precision when providing substitutions for lay terms, abbreviations, and acronyms. We have found qualitative evidence that reformulating queries with professional terminology may be a promising strategy to improve consumer health-information searches, although we caution that automated reformulation could in fact worsen search performance when the terminology is ill-fitted or arcane.

## Introduction

An ever-increasing number of patients and their family members are turning to the Internet for health information [1]. Recent survey reports suggest that at least half of the adults in the United States have searched for health information online [2]. Careful analysis of consumer information needs and preferences through the field of consumer health informatics is increasingly important to ensure that the information retrieval process is positive and effective [3]. Specifically, studying the variably effective search strategies and their associated performance could provide valuable insight for the development of future consumer health-information retrieval tools.

At present, it appears that most people who search the Internet are not using the most effective strategies. Spink et al analyzed one million queries from the log data of a popular Internet search engine and found that most people used short (mean 2.4 terms) and unmodified queries [4]. The one billion queries analyzed by Silverstein et al had a similarly small mean number of terms (2.35) [5]. Further, 77% of these search sessions consisted of just one query; this means a small minority of searchers modified their query after the first search. Although it cannot be ascertained what percentage of the logged searches were performed by humans rather than search robots, it is likely safe to conclude that Internet searchers are using short and therefore imprecise queries. Also, searchers are likely to quit after one search iteration instead of modifying their search to improve the results. Observational studies have shown that consumers specifically searching for health-care information employ the same suboptimal search strategies [6-8].

One potential tactic to address the problem of short and imprecise queries is to automatically alter the initial query for better returns, either by reformulation or expansion. Previous research, including a study that mapped consumer terms to an established medical vocabulary, has shown that there is a significant mismatch between consumers' health vocabulary and the terminology of the content [8]. Tse and Soergel's review of postings to online health discussion forums showed that a majority of consumer terms, although overlapping with professional terminology conceptually, often do not take the same form as technical terms [9]. This mismatch of consumer and medical content terminology could be partially bridged using query expansion, which has been shown to improve search performance both inside [10] and outside [11,12] the medical domain. Search behavior research has also demonstrated the difficulty that end-users have selecting query terms and illustrates the potential benefit of providing a thesaurus to suggest alternative queries and improve search [13,14]. Similarly, we theorize that reformulating queries to replace lay terms with the terminology more commonly used in medical content could potentially facilitate the delivery of relevant content to consumers.

To investigate the effect of query reformulation using standardized medical terminologies, we utilized original consumer health-information queries with explicit information needs and the Unified Medical Language System (UMLS) Metathesaurus [15], as the terminology source. We collected our search queries through interviews with consumers, and were thus able to ascertain the specific intention that led to the resulting free-text queries. This enabled a more objective assessment of the success of a given search. We studied the effect of reformulation in two different search spaces: the broad scope of a commercial search engine and the more limited scope of a single consumer health-information site. Survey data [16] and research [2,6] have shown that most consumers initiate their search for health information using a general search engine. However, although more limited in content, the information provided by a consumer information source like MedlinePlus [17] is of a more consistent quality, and the site receives 2 million queries per month by our own calculation of its log data. We were therefore interested in the effect of query reformulation in both of these settings. The research question addressed in this study is whether reformulating original consumer queries with preferred terminology from UMLS Metathesaurus lead to better search results.

## Methods

### Collection of Consumer Queries and Search Goals

Consumer queries and search goals were collected through an ongoing study we were conducting with patients and visitors recruited from public areas of Brigham and Women's Hospital, a large teaching hospital in Boston. Subjects described their health-information needs to the interviewer (RP). Each participant was then given the opportunity to search the Internet on a laptop computer to find the answer to his or her specific question or questions. The free-text queries generated by participants for these searches were recorded for further analysis. Search goals were recorded by the researcher based on interviews with the consumers.

### Selecting Queries for Further Testing

Suitable substitutions for user-generated queries were generated using the UMLS Metathesaurus (release 2003AB). The Metathesaurus stores information about biomedical concepts compiled from numerous vocabularies and sources. Synonyms and inter-concept relationships are among the many attributes recorded for each concept, with one term chosen as the preferred English name for each concept. In this study, the search queries generated by consumers were hand-mapped to Metathesaurus-preferred concept names. For example, the consumer query "stroke" was deemed to be a synonym of the Metathesaurus concept "cerebrovascular accident." Some of the consumers' queries (eg, "chronic pain") were identical to the primary term used in the Metathesaurus. Only queries that were not equivalent to Metathesaurus preferred terms, and that therefore could be reformulated, were selected for this study.

### Gold Standard Answer Generation

For each consumer question, a gold standard answer specific to the consumers' information needs was generated by an investigator with medical training (RP). Harrison's Online and MDConsult were the main resources used to create these answers. Gold standard answers were used to assess and compare the results generated from the Internet searches conducted for this study.

### Query Reformulation

User queries were mapped to concepts in the UMLS Metathesaurus. Only queries that had at least one term found to be in the list of synonyms for a preferred concept name were selected for reformulation. Queries were reformulated by replacing the user term with the preferred synonym. Terms within queries that already corresponded to preferred concepts were left unaltered. For instance, the word "thyroid" was unchanged in the reformulation of the user query "thyroid abs test." Only one concept name was altered at a time. User queries that contained n-terms that could be mapped to preferred concepts thus led to the generation of n-reformulations, each with one user term replaced by the corresponding professional phrase. For example, two reformulations were generated from the user query "herbal treatment cancer": "herbal therapeutic aspects cancer" and "herbal treatment malignant neoplasms."

Query reformulation (altering the initial query), rather than query expansion (adding synonyms to the initial query), was the methodology chosen for this study. Metathesaurus-based query expansion has been shown to cause a decline in search performance [18]. Imprecise or arcane synonyms such as "blastoma, NOS" for the concept "malignant neoplasms" and "apoplectic fit" for the concept "cerebrovascular accident" could dilute the original intention of a given query and decrease retrieval performance.

### Internet Search Using Original Consumer Queries and Reformulated Queries

Both the original consumer-generated queries and the reformulated queries were used to initiate controlled searches in two different search spaces: the more health-specific MedlinePlus and the broader range of content covered by a Google search.

MedlinePlus is a high-quality consumer-health site provided by the National Library of Medicine. The continuously updated content of this noncommercial site, organized by health topic, includes information on over 600 diseases and conditions, as well as a medical encyclopedia and information on prescription drugs [17]. Links to additional resources from the National Institutes of Health and other trusted sources are also presented.

Google [19] is currently a leading search engine. It provides access to over 3 billion indexed Web pages. Its proprietary search algorithm ranks the relevance of Web pages based in part on the number of links made to the page from other sites, and on characteristics of the page itself. The authority of referring pages is also considered in determining the rank of a page [20]. Among advanced search features available is the ability to limit searches using the Google engine to specific Web sites; this feature was employed for our study.

We used both the consumers' original queries and the modified queries to conduct Internet searches. Quotation marks were placed on each end of the query text (eg, the search query for flat head was "flat head"). The Google search engine was used to search both the consumer health-oriented site (MedlinePlus) and the general Internet (Google). Searches using the search engine included on the MedlinePlus site yielded several lists of results organized by health topic, whereas Google-initiated searches yielded a single list ordered by relevance. The Google search engine was utilized so that search results would be identically formatted and therefore more suited for comparison. Every search was limited to English language pages using Google's "Advanced Search" language feature.

Standard information, such as the date and the total number of results, was recorded for every search. The first 30 hits of every search, the number of documents that users are reasonably willing to look at after a search [18], were assessed for the presence of the gold standard answer to the participant's original question. A result page was considered to contain the gold standard answer if

1. the answer could be found by following no more than one link from the initial page
2. at least 90% of the established gold standard answer was present (for questions whose answers were lists, such as stroke risk factors)
3. for questions whose goal was to obtain general information about a topic, the page contained at least one correct fact pertinent to the health topic.

The total number of assessed hits containing the gold standard answer was recorded and the fraction of the assessed hits containing the gold standard answer (out of no more than 30) was calculated. This figure was used as an estimate of precision and was used for comparison of searches. Queries that returned no result or results that contained no gold standard answer failed to satisfy users' information needs, although we do recognize that there can be true negatives and returning results that do not contain the right information may misinform users and cost time to process. For the convenience of performance comparison of all queries, when no result was returned, the top 30 precision was assigned 0 in this study. True negative rates, however, are examined for those queries that failed to return any result. To determine true negative rates, the investigators conducted numerous searches of MedlinePlus and Google and browsed the concept-specific content of MedlinePlus to search for the gold standard answer. Substitutions that seemed to improve results versus those that did not were examined for reasons. Additionally, the queries and search results were examined for general qualitative trends.

## Results

**Figure 1:**
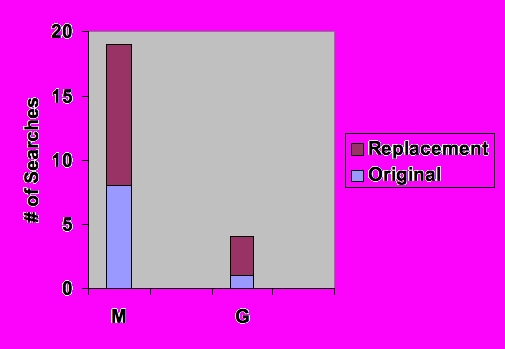
Distribution of searches with no returns (n=23) (M = MedlinePlus, G = Google)

A total of 16 queries were selected for substitution from an initial pool of 46 queries. Eighteen replacement queries were generated. The original queries and their substitutions are summarized in Table 1. Each query (consumer or reformulation) was used to conduct two Internet searches, for a total of 68 searches: 34 in MedlinePlus and 34 in Google. In all, 926 individual search result pages were examined. Of the 68 searches, 23 did not yield any results. The text of these queries is listed in Table 2, and the distribution of the searches in MedlinePlus and Google is illustrated in Figure 1. Nine of these empty searches were generated using the consumers' original queries and 14 were based on reformulated queries; 19 were in MedlinePlus and 4 were in Google. In other words, using original queries in Google resulted in the least number of searches (only 1) with no returns. To put this result into perspective, we found that MedlinePlus contained the gold standard answer for 15 of the 19 failed queries (79%), and Google contained the answer for all 4 of the searches that had no returns (100%). In other words, among the queries that did not return any result, the true negative rate is 21% for MedlinePlus and 0% for Google.

**Table 1:** Consumer queries (n=16) with reformulated queries (n=18) and search goals

**Participant's Question**	**Query Text** *(reformulated queries in italics)*	**Precision of Search** *0 - No retrieved documents with gold standard answer* *Ø - No retrieved documents*
		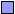 MedlinePlus 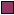 Google
Are there any natural substitutes for the hormone replacement therapy agent Prempro?	natural alternative hrt *natural alternative hormone replacement therapy*	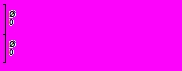
	natural hrt *natural hormone replacement therapy*	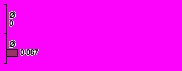
Are there support groups for restless legs syndrome?	restless leg syndrome *restless legs syndrome*	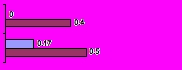
General information about heart transplants	heart transplant *heart transplantation*	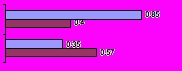
General information about petit mal seizures	petit mal seizure *epilepsy, absence*	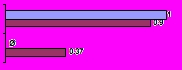
General information about plagiocephaly	flat head *plagiocephaly*	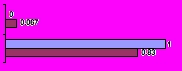
How are arrhythmias treated?	heart arrhythmia treatment *arrhythmia treatment* *heart arrhythmia therapeutic aspects*	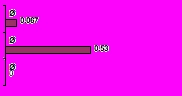
How are the results of the anti-TPO thyroid lab test interpreted?	thyroid abs test *thyroid antibody studies*	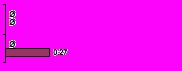
How do problems with the heart's electric system lead to shortness of breath?	heart electric *heart conduction system*	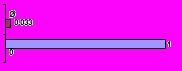
Is there treatment for restless legs syndrome?	restless leg syndrome *restless legs syndrome*	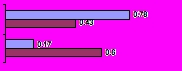
What are scientifically validated treatments for cancer?	herbal treatment cancer *herbal therapeutic aspects cancerherbal treatment malignant neoplasms*	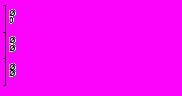
What are the risk factors for stroke?	stroke *cerebrovascular accident*	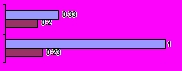
What are the side effects of Lexapro?	ssri *selective serotonin re-uptake inhibitor*	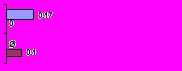
What causes heart flutters (palpitations)?	heart flutters *fluttering heart*	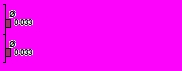
What foods should be avoided to prevent cavities in children?	cavity *dental caries*	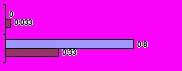
Why can't you have an MRI with a pacemaker?	contraindications mri *contraindications magnetic resonance imaging*	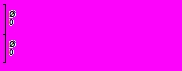

**Table 2:** Searches that did not yield any returns (n=23) G signifies search in Google, and M indicates a search of MedlinePlus

**Query Text**	**Original (O) or Replacement (R)?**	**Search Scope**
"contraindications mri"	O	M
"heart arrhythmia treatment"	O	M
"heart electric"	O	M
"heart flutters"	O	M
"herbal treatment cancer"	O	M
"natural alternative hrt"	O	M
"natural hrt"	O	M
"thyroid abs test"	O	M, G
"arrhythmia treatment"	R	M
"contraindications magnetic resonance imaging"	R	M
"epilepsy, absence"	R	M
"fluttering heart"	R	M
"heart arrhythmia therapeutic aspects"	R	M, G
"herbal therapeutic aspects cancer"	R	M, G
"herbal treatment malignant neoplasms"	R	M, G
"natural alternative hormone replacement therapy"	R	M
"natural hormone replacement therapy"	R	M
"selective serotonin re-uptake inhibitor"	R	M
"thyroid antibody studies"	R	M

**Figure 2:**
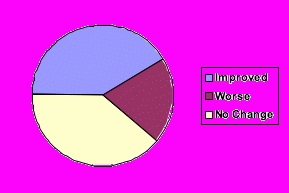
Performance of reformulated queries (n = 36)

The main measure of the success of a search was the percentage of the first 30 resulting pages that contained the gold standard answer (top 30 precision). For example, 10 of the first hits of the MedlinePlus search for "stroke" contained enough of the established stroke risk factors to qualify as gold standard. The search had precision of 33% (10/30). By contrast, the Google search for "thyroid antibody studies" yielded only 11 hits. Of these 11, 3 contained the requested information regarding the anti-TPO lab test. This search therefore had a precision of 27% (3/11). As mentioned previously, when a query generated no result, its top 30 precision was set to 0. The precision values for all 68 searches (mean = 0.22, SD = 0.32) are presented in Table 1. The mean precision for all searches in MedlinePlus was 0.23 (SD = 0.37) and the mean precision for searches in Google was 0.21 (SD = 0.26).

Using precision as a performance indictor, 15 of the 36 searches using reformulated queries yielded better returns than their associated original queries (5 in MedlinePlus and 10 in Google). Seven of the searches using reformulated queries showed a worse performance than the original substitution as indicated by precision (4 MedlinePlus and 3 Google). The performance of the remaining 14 searches was unchanged by reformulation. Table 3 shows how the individual queries performed, and Figure 2 summarizes the relative performance of the reformulated queries.

**Table 3:** Search performance after reformulation. Original queries are listed either as improved, worse, or no change with substitution. G signified search in Google and M indicates a search of MedlinePlus

**Query Text**	**Replacement Text**	**Search Space(s)**
**Improved with Reformulation**		
"cavity"	"dental caries"	G, M
"flat head"	"plagiocephaly"	G, M
"heart arrhythmia treatment"	"arrhythmia treatment"	G
"heart electric"	"heart conduction system"	M
"heart transplant"	"heart transplantation"	G
"natural hrt"	"natural hormone replacement therapy"	G
"restless leg syndrome"	"restless legs syndrome"	G, M
"restless leg syndrome"	"restless legs syndrome"	G
"ssri"	"selective serotonin re-uptake inhibitor"	G
"stroke"	"cerebrovascular accident"	G, M
"thyroid abs test"	"thyroid antibody studies"	G

**Worse with Reformulation**		
"heart electric"	"heart conduction system"	G
"heart transplant"	"heart transplantation"	M
"petit mal seizure"	"epilepsy, absence"	G, M
"restless leg syndrome"^2^	"restless legs syndrome"	M
"heart arrhythmia treatment"	"heart arrhythmia therapeutic aspects"	G
"ssri"	"selective serotonin re-uptake inhibitor"	M

**No Change with Reformulation**		
"contraindications magnetic resonance imaging"	"contraindications mri"	G, M
"heart arrhythmia treatment"	"arrhythmia treatment"	M
"heart arrhythmia treatment"	"heart arrhythmia therapeutic aspects"	M
"heart flutters"	"fluttering heart"	G, M
"herbal treatment cancer"	"herbal therapeutic aspects cancer"	G, M
"herbal treatment cancer"	"herbal treatment malignant neoplasms"	G, M
"natural alternative hrt"	"natural alternative hormone replacement therapy"	G, M
"natural hrt"	"natural hormone replacement therapy"	M
"thyroid abs test"	"thyroid antibody studies"	M

Three reasons for changes in retrieval performance (precision) were identified: First, several consumer searches using ambiguous lay terms were improved when reformulated with professional terminology. This trend was noted both in MedlinePlus and in Google. Second, searches based on queries utilizing acronyms were improved in the Google scope when they were expanded to full phrases. Third, certain queries containing professional terms that were arcane or contextually ill-fitted to the users' original search goals performed worse than the original queries.

## Discussion

### Significance and Implication

Conducting Internet searches with reformulated consumer queries allowed us to note qualitative trends in query reformulation with professional terminology: it often helped to improve query performance by reducing ambiguity and increasing distinguishing power, but sometimes reduced query performance when the professional terms were arcane or ill-fitted. The reformulated queries also often had no impact on performance. Approximately 35% of the consumer queries we collected did not use UMLS-preferred names for concepts and were thus suitable for reformulation. This represents a substantial portion of the original sample queries that could potentially be affected by the reformulation approach.

#### Benefit of Query Reformulation

In 15 of 36 instances, the replacement queries yielded better results than the originals (as indicated by top 30 precision and taking into account the queries that did not generate any results). Searches using queries that utilized ambiguous lay terms such as "cavity," "flat head," and "stroke," were improved when replaced with professional terms ("dental caries," "plagiocephaly," and "cerebrovascular accident," respectively). These searches improved in both the health-specific scope of MedlinePlus and the broader Google domain. One participant used the ambiguous query "flat head" to search for information about plagiocephaly (infant cranial asymmetry). Many of the sites listed after the "flat head" search in Google utilized non-medical interpretations of the phrase (screwdrivers, screws, and even a guitar). In fact, only 2 of the first 30 hits contained contextually appropriate information. The search for "plagiocephaly," however, yielded better results than the ambiguous term "flat head": 25 of 30 hits were contextually correct.

It is important to note that many medical sites do employ lay terms. However, professional terms tend to have better distinguishing power in locating medical contents. For example, many of the "plagiocephaly" pages contained the phrase "flat head," while sites about screwdrivers or guitars do not contain the word "plagiocephaly." These medical sites using "flat head" were clearly outnumbered by pages using "flat head" in a non-medical context, and were therefore almost entirely absent from the "flat head" Internet search.

Acronyms or abbreviations are likely to introduce ambiguity to queries and thus can benefit from reformulation. Searches composed of acronyms or initialisms (eg, "SSRI" for selective serotonin reuptake inhibitor and "HRT" for hormone replacement therapy) or abbreviations (eg, "abs" for antibodies) fared better when reformulated with the full phrase. This trend was noted only in the broader Google domain. This is not surprising because there is a much greater chance of the existence of a non-medical meaning of these short terms in the broader scope of Google than in the exclusively medical scope of MedlinePlus. For instance, in addition to being a drug class, SSRI is also a pop band, an institute, and a stock abbreviation. Links interpreting SSRI in all of these ways were found in the first 30 Google search results. The search for "selective serotonin re-uptake inhibitor," by contrast, eliminated pages with these alternatives. The MedlinePlus search for "selective serotonin re-uptake inhibitor," however, yielded no results, due in part to the lack of an exact text match with the hyphenated spelling of the work "reuptake." Removing the ambiguity of acronyms and abbreviations from queries improved the performance of Internet searches conducted with the general search engine.

#### Disadvantage of Query Reformulation

In 7 of 36 instances, search performance was worse when the original consumer queries were replaced with alternate phrases. Four of these were conducted in the MedlinePlus domain, and three were in Google. It is not surprising that one of these searches, "petit mal seizure," performed worse when replaced with "epilepsy, absence," the Metathesaurus-preferred term. The consumer's question was about a medical event, a seizure, and the reformulated query referred to the disease that causes the event. Although altered less dramatically, the queries "heart transplant" and "restless leg syndrome" also performed considerably worse when reformulated. The arcane term "therapeutic aspects" replaced "treatment" in the query "heart arrhythmia treatment," which contributed to a decrease in precision in the Google search. We are aware of other concepts, not among the 16 consumer queries, with arcane preferred names in UMLS. For instance, "pes," is the Metathesaurus preferred term for "foot." These examples illustrate that an automated query-replacement process would have the potential for flawed substitutions. Presenting a search term that produces worse results than an information seeker's original query could lead to great frustration for the seeker.

#### Content Scope and Quality

The role of query reformulation appears to be more significant for a large content scope that for a health-care specific site simply because there is more room for ambiguity when the scope is extremely large. However, an aspect that we did not measure in this study is the rate of misinformation. Combing through almost 1000 sites allowed for general observations about the reliability and quality of the information presented to consumers after an Internet search. Using a general search engine like Google, we encountered a great number of sites with misleading or biased information. For instance, the Google search for "natural hormone replacement therapy" resulted in several pages selling "cures" for aging. Similarly, Web sites promoting alternative therapies for cancer have been found to be of dubious quality [21].

Unfortunately, searching within the domain of a single high-quality consumer site is not without disadvantages. Because a single site contains a small fraction of the information available on the general Internet, the chance of a finding the desired information is diminished. In this study, for example, although all searches were conducted in both MedlinePlus and Google, there were twice as many searches with at least one result in Google (n=30) as in MedlinePlus (n=15). The true negative rate for MedlinePlus (21%) was considerably higher than that for Google (0%), further emphasizing that medical sites, though providing a more consistent quality of information, will not contain the answer a percentage of consumers' queries. We have written a manuscript comparing medically specific and general search scopes, which discusses the pros and cons of each based on a separate study. Resolving the trade-off between quality and breadth of information remains a major challenge to successful consumer information retrieval.

#### Advantages to Queries With Known Goals

There are advantages to assessing health-related Web searches in this manner. The information needs of the consumer are explicit because they have been obtained from direct interviewing. When queries are obtained from log data, the intentions of the consumer are open to conjecture. The information needs of the query "flat head," for example, would prove difficult to guess without the context of an interview. Although many of the users had very precise goals, the queries they formulated contained little more than the name of the disease or condition in question. In one search, the participant had a question specific to the medication Lexapro but did not even include the drug name in the search. Without the interview, it would be impossible to guess that the consumer searching with the query "restless leg syndrome" was specifically looking for pharmacologic treatment of that condition. These observations provide further evidence to the reported observation that consumers are producing short, imprecise queries. The type of detailed analysis employed in this study provides a more precise picture of the needs of health-information seekers in the hope of facilitating well-informed system development.

### Limitations

The relatively small number of available sample queries with known goals limited the analysis to qualitative review instead of a statistically significant quantitative measure of search precision. The numbers of queries available for reformulation was further limited by the requirement that the original term not be a Metathesaurus preferred concept name. Not surprisingly, many participants used preferred concept drug and disease names for their initial searches. Further, even with a direct interview, some of the participants' information needs could not be expounded beyond the general desire for more information.

The gold standard answer presents another limitation. Because each site presents information differently, it is not possible to apply identical standards from site to site. This form of assessment, though time-consuming, is far more detailed than merely searching for the presence of the term.

Using quotation marks for queries was a major factor contributing to the number of searches that did not return results. We chose to utilize quoted query phrases so we could assess the impact of the phrase as a whole rather than the individual words, which each play a separate part in the search when not contained by quotation marks.

The method used to search MedlinePlus had one further limitation. This site was searched using Google instead of the search engine included with the site. The search results are not the same when the MedlinePlus search mechanism is used instead of Google. We did not intend to assess the performance of MedlinePlus but rather to study the impact of query reformulation in the narrower and specifically medical scope of a consumer health-information site as well as the much broader swath of the Internet covered by Google.

### Further Study

We are in the process of

1. expanding our database of user queries with known intent
2. conducting a study with similar methods of patients in an asthma center to assess the specific information needs and search strategies of a specific health consumer population
3. developing a search tool that provides suggested search queries based on the initial search entered by a user.

### Conclusion

We investigated the effect of reformulating consumer health queries using professional terminology. This study has shown some qualitative evidence that reformulating queries with professional terminology may be a promising strategy to improve consumer health-information searches. After taking original queries with clearly defined goals from health-information consumers and replacing the search text with phrases from medical vocabulary, we noted a trend towards increased precision when providing substitutions for lay terms, abbreviations, and acronyms. This improvement was noted both in searches conducted in the narrower scope of a consumer health site and in searches of a much broader portion of the Internet using the popular search engine Google. We caution, however, that automated reformulation could in fact worsen search performance when the terminology is ill-fitted or arcane.

## Abbreviations

UMLS: Unified Medical Language System
